# The genome sequence of the Streak, *Chesias legatella* (Denis & Schiffermüller, 1775)

**DOI:** 10.12688/wellcomeopenres.19298.1

**Published:** 2023-05-10

**Authors:** David Lees, Natural History Museum Genome Acquisition Lab, Darwin Tree of Life Barcoding collective, Wellcome Sanger Institute Tree of Life programme, Wellcome Sanger Institute Scientific Operations: DNA Pipelines collective, Tree of Life Core Informatics collective, Darwin Tree of Life Consortium

**Affiliations:** 1 Natural History Museum, London, England, UK

**Keywords:** Chesias legatella, the Streak, genome sequence, chromosomal, Lepidoptera

## Abstract

We present a genome assembly from an individual male *Chesias legatella* (the Streak; Arthropoda; Insecta; Lepidoptera; Geometridae). The genome sequence is 310.3 megabases in span. Most of the assembly is scaffolded into 31 chromosomal pseudomolecules, including the Z sex chromosome. The mitochondrial genome has also been assembled and is 20.1 kilobases in length.

## Species taxonomy

Eukaryota; Metazoa; Ecdysozoa; Arthropoda; Hexapoda; Insecta; Pterygota; Neoptera; Endopterygota; Lepidoptera; Glossata; Ditrysia; Geometroidea; Geometridae; Larentiinae; *Chesias*; *Chesias legatella* (Denis & Schiffermüller, 1775) (NCBI:txid934925).

## Background

The Streak, *Chesias legatella*, is a medium sized geometrid moth, dark greyish brown with a prominent creamish-white streak towards the apex of the forewing and an elliptic dark shape in the discal area with another whitish-cream dash inside, which flies late in the temperate season, usually September to early November in the UK ( Randle *et al.*, 2019), overwintering as an egg. At rest it has a rather unusual posture for a looper moth, sometimes rolling its wings partially around a twig.

The Streak is a species of open woodland and heathland in the UK, especially on sandy substrates, feeding on Broom ( *Cytisus scoparius* L.) ( Wall, 1975), or occasionally Tree Lupin ( *Lupinus arboreus*) ( Wall, 1975; Waring *et al.*, 2017).

*C. legatella* is generally common and widespread in the western Palaearctic only, from southern Scandinavia to the northern Mediterranean; but has relatively few records for eastern Europe ( GBIF Secretariat, 2022). It is widespread in the UK and eastern Ireland ( NBN Atlas Partnership, 2021), but the distribution is patchy, and it is vulnerable, with evidence for a significant decline since 1970 ( Conrad *et al.*, 2006) that has affected its distribution ( Randle *et al.*, 2019).

There is a single DNA barcode cluster on BOLD, BOLD:AAF2574 (16 March 2023), which is 5.46% pairwise divergent to that of *Chesias capriata* Prout, 1904 from Italy (BOLD:AAW3724). *C. legatella* has six other known congeners including the Broom-tip *C. rufata* (Fabricius, 1775) and belongs to the larentiine tribe Chesiadini, an early diverging one within the subfamily Larentiinae (after Trichopterygini), based on a study of ten nuclear protein coding genes and COI ( Murillo-Ramos *et al.*, 2019: Figure 3). The genus *Chesias* falls sister to the genus *Aplocera* Stephens, 1827 in the study of Õunap, Viidalepp and Truuverk (2016: Figure 2). The whole genome will be useful for more detailed evolutionary studies.

The species is of no economic concern, although it has been considered as a possible agent of biological control of Broom ( Syrett *et al.*, 1999), probably without consequence.

The genome of *Chesias legatella* was sequenced as part of the Darwin Tree of Life Project, a collaborative effort to sequence all named eukaryotic species in the Atlantic Archipelago of Britain and Ireland. Here we present a chromosomally complete genome sequence for *Chesias legatella*, based on one specimen from Beinn Eighe National Nature Reserve, Scotland.

## Genome sequence report

**Figure 1.**
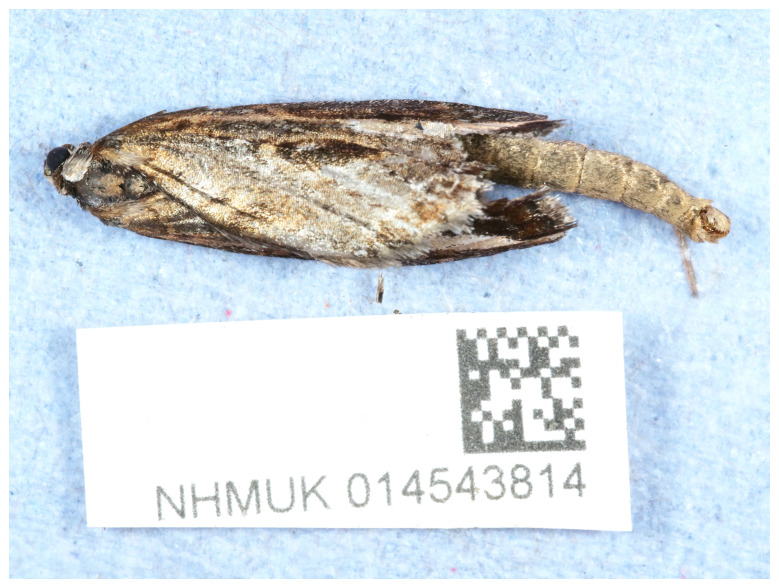
Photograph of the *Chesias legatella* (ilCheLega1) specimen used for genome sequencing.

The genome was sequenced from one male of *Chesias legatella* ( Figure 1) collected from Beinn Eighe National Nature Reserve, Scotland, UK (latitude 57.63, longitude –5.35). A total of 53-fold coverage in Pacific Biosciences single-molecule HiFi long reads was generated. Primary assembly contigs were scaffolded with chromosome conformation Hi-C data. Manual assembly curation corrected seven missing or mis-joins and removed two haplotypic duplications, reducing the scaffold number by 5.26%.

**Figure 2.**
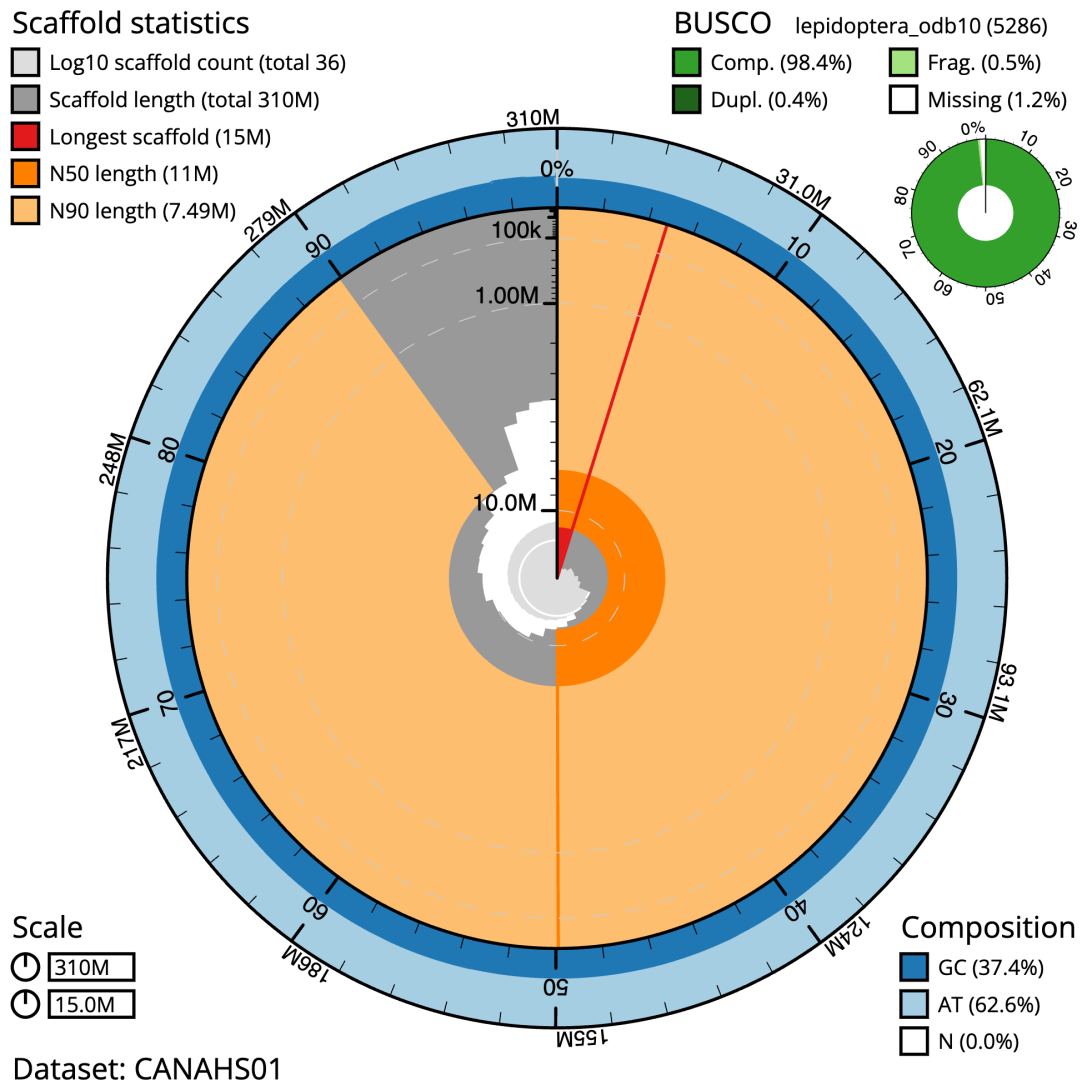
Genome assembly of *Chesias legatella*, ilCheLega1.1: metrics. The BlobToolKit Snailplot shows N50 metrics and BUSCO gene completeness. The main plot is divided into 1,000 size-ordered bins around the circumference with each bin representing 0.1% of the 310,278,188 bp assembly. The distribution of scaffold lengths is shown in dark grey with the plot radius scaled to the longest scaffold present in the assembly (14,956,362 bp, shown in red). Orange and pale-orange arcs show the N50 and N90 scaffold lengths (11,018,016 and 7,493,246 bp), respectively. The pale grey spiral shows the cumulative scaffold count on a log scale with white scale lines showing successive orders of magnitude. The blue and pale-blue area around the outside of the plot shows the distribution of GC, AT and N percentages in the same bins as the inner plot. A summary of complete, fragmented, duplicated and missing BUSCO genes in the lepidoptera_odb10 set is shown in the top right. An interactive version of this figure is available at https://blobtoolkit.genomehubs.org/view/ilCheLega1.1/dataset/CANAHS01/snail.

The final assembly has a total length of 310.3 Mb in 36 sequence scaffolds with a scaffold N50 of 11.0 Mb ( Table 1). Most (99.95%) of the assembly sequence was assigned to 31 chromosomal-level scaffolds, representing 30 autosomes, and the Z sex chromosome. Chromosome-scale scaffolds confirmed by the Hi-C data are named in order of size ( Figure 2– Figure 5; Table 2). While not fully phased, the assembly deposited is of one haplotype. Contigs corresponding to the second haplotype have also been deposited.

**Table 1.** Genome data for *Chesias legatella*, ilCheLega1.1.

Project accession data		
Assembly identifier	ilCheLega1.1	
Species	*Chesias legatella*	
Specimen	ilCheLega1	
NCBI taxonomy ID	934925	
BioProject	PRJEB55725	
BioSample ID	SAMEA14448143	
Isolate information	ilCheLega1, head and thorax (genome sequencing and Hi-C scaffolding)	
Assembly metrics *		*Benchmark*
Consensus quality (QV)	65.8	*≥ 50*
*k*-mer completeness	100%	*≥ 95%*
BUSCO **	C:98.4%[S:98.0%,D:0.4%], F:0.5%,M:1.2%,n:5,286	*C ≥ 95%*
Percentage of assembly mapped to chromosomes	99.95%	*≥ 95%*
Sex chromosomes	Z chromosome	*localised homologous pairs*
Organelles	Mitochondrial genome assembled	*complete single alleles*
Raw data accessions		
PacificBiosciences SEQUEL II	ERR10168717	
Hi-C Illumina	ERR10149547	
Genome assembly		
Assembly accession	GCA_947359385.1	
*Accession of alternate haplotype*	GCA_947359375.1	
Span (Mb)	310.3	
Number of contigs	80	
Contig N50 length (Mb)	6.2	
Number of scaffolds	36	
Scaffold N50 length (Mb)	11.0	
Longest scaffold (Mb)	15.0	

* Assembly metric benchmarks are adapted from column VGP-2020 of “Table 1: Proposed standards and metrics for defining genome assembly quality” from ( Rhie *et al.*, 2021). ** BUSCO scores based on the lepidoptera_odb10 BUSCO set using v5.3.2. C = complete [S = single copy, D = duplicated], F = fragmented, M = missing, n = number of orthologues in comparison. A full set of BUSCO scores is available at https://blobtoolkit.genomehubs.org/view/ilCheLega1.1/dataset/CANAHS01/busco.

**Figure 3.**
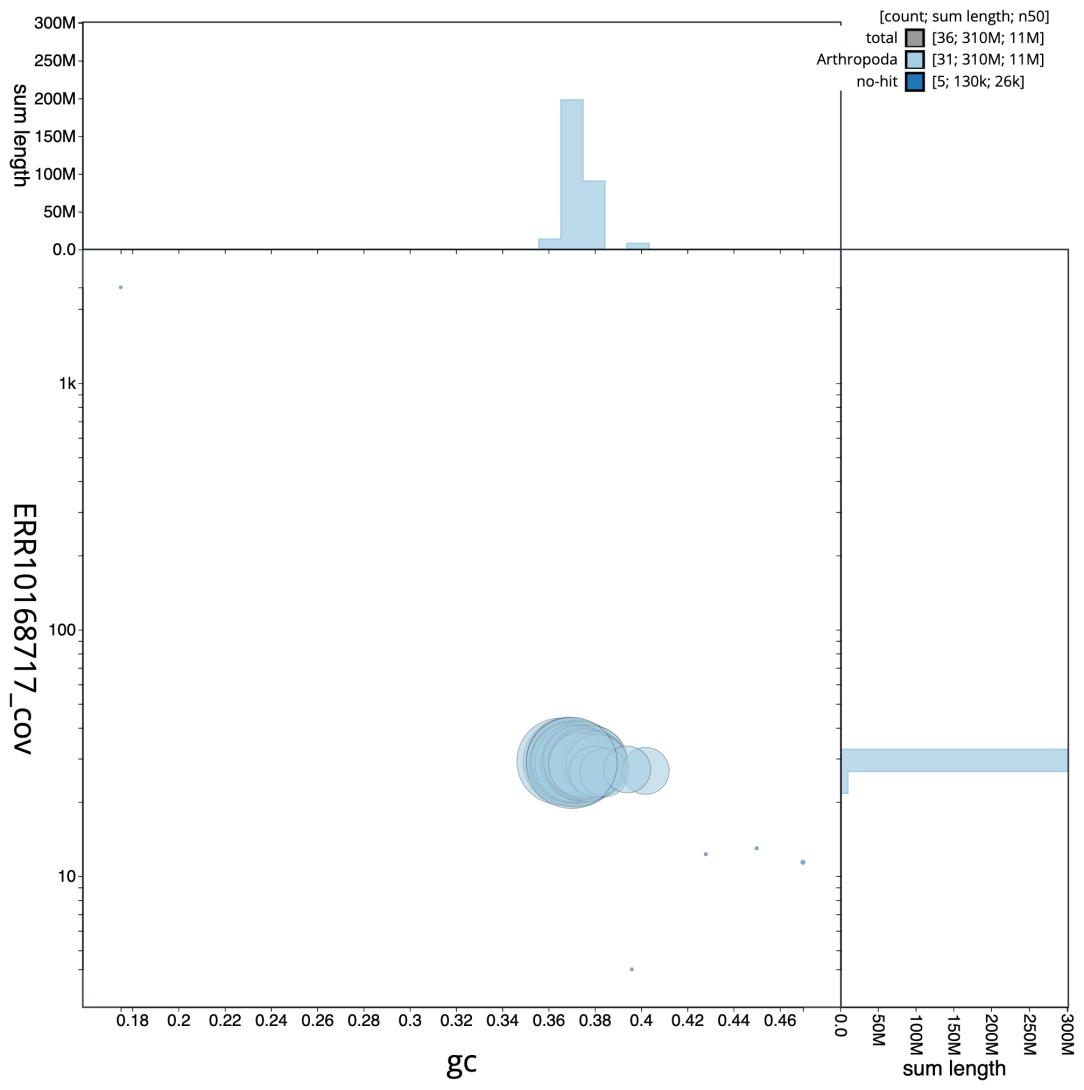
Genome assembly of *Chesias legatella*, ilCheLega1.1: BlobToolKit GC-coverage plot. Scaffolds are coloured by phylum. Circles are sized in proportion to scaffold length. Histograms show the distribution of scaffold length sum along each axis. An interactive version of this figure is available at https://blobtoolkit.genomehubs.org/view/ilCheLega1.1/dataset/CANAHS01/blob.

**Figure 4.**
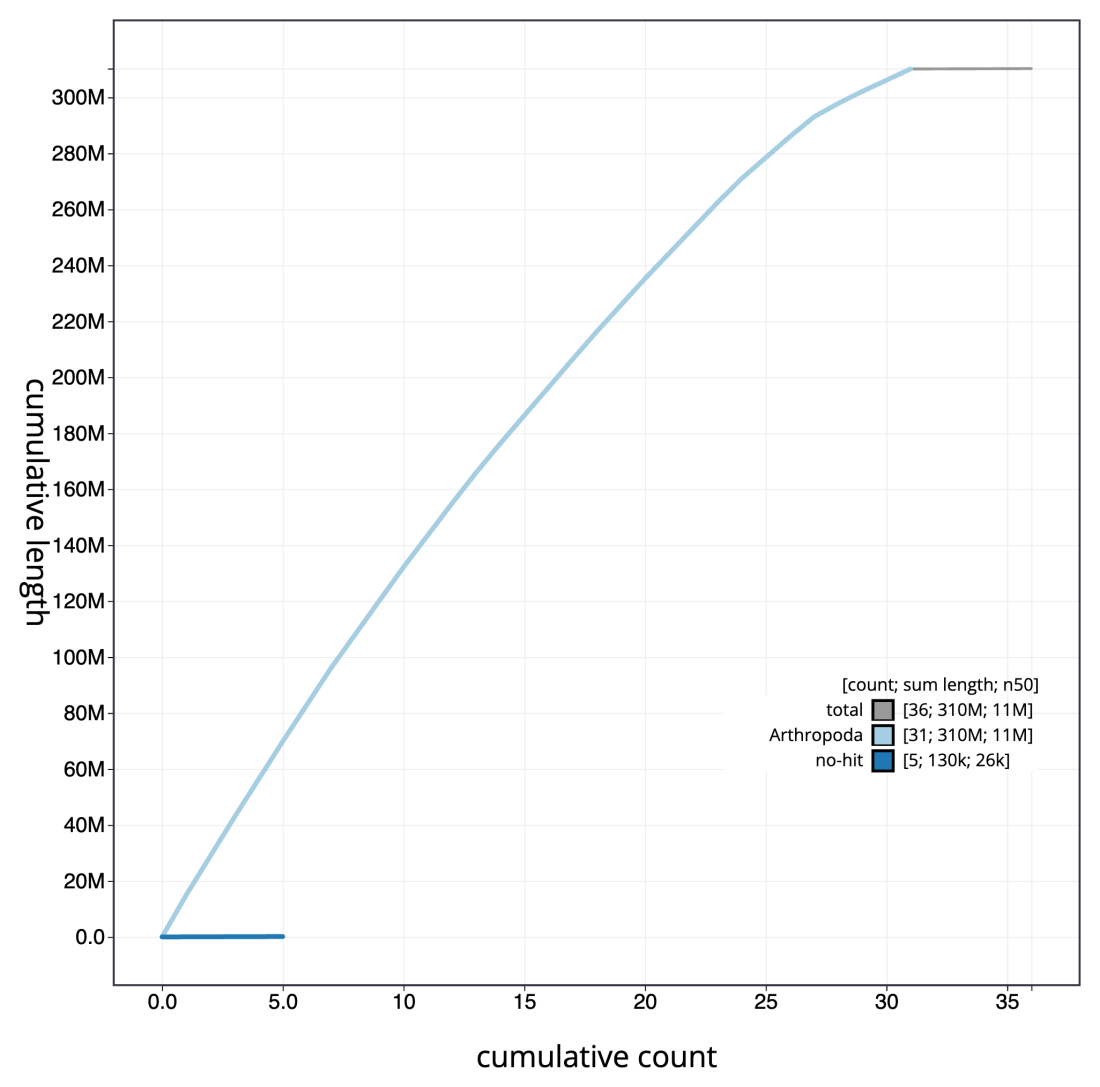
Genome assembly of *Chesias legatella*, ilCheLega1.1: BlobToolKit cumulative sequence plot. The grey line shows cumulative length for all scaffolds. Coloured lines show cumulative lengths of scaffolds assigned to each phylum using the buscogenes taxrule. An interactive version of this figure is available at https://blobtoolkit.genomehubs.org/view/ilCheLega1.1/dataset/CANAHS01/cumulative.

**Figure 5.**
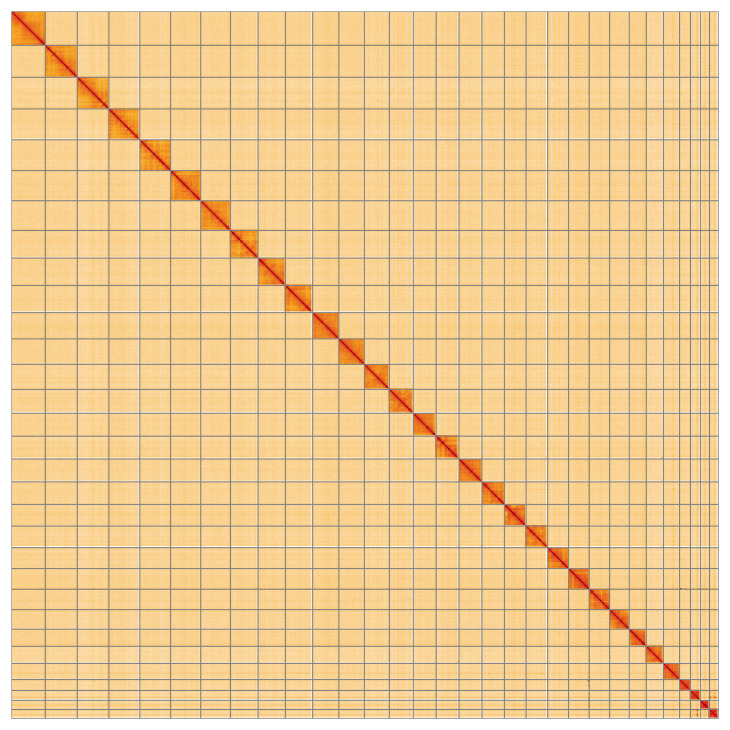
Genome assembly of *Chesias legatella*, ilCheLega1.1: Hi-C contact map of the ilCheLega1.1 assembly, visualised using HiGlass. Chromosomes are shown in order of size from left to right and top to bottom. An interactive version of this figure may be viewed at https://genome-note-higlass.tol.sanger.ac.uk/l/?d=ZLBbPbLmQzijgU5VCAKOxg.

**Table 2.** Chromosomal pseudomolecules in the genome assembly of *Chesias legatella*, ilCheLega1.

INSDC accession	Chromosome	Size (Mb)	GC%
OX375763.1	1	14.01	36.8
OX375764.1	2	13.87	37.2
OX375765.1	3	13.53	37.1
OX375766.1	4	13.5	36.5
OX375767.1	5	13.22	36.9
OX375768.1	6	13.02	37
OX375769.1	7	12.11	37
OX375770.1	8	12.02	37.1
OX375771.1	9	11.91	37
OX375772.1	10	11.51	37.4
OX375773.1	11	11.17	37.3
OX375774.1	12	11.02	37.1
OX375775.1	13	10.42	37.5
OX375776.1	14	10.06	37.3
OX375777.1	15	10.02	37.8
OX375778.1	16	10.02	37.8
OX375779.1	17	9.91	37.4
OX375780.1	18	9.47	37.5
OX375781.1	19	9.47	37.7
OX375782.1	20	9.15	37.4
OX375783.1	21	9.1	37.3
OX375784.1	22	8.91	37.5
OX375785.1	23	8.57	37.5
OX375786.1	24	7.53	38
OX375787.1	25	7.49	37.4
OX375788.1	26	7.07	38.1
OX375789.1	27	4.79	38
OX375790.1	28	4.31	38.4
OX375791.1	29	4.02	40.2
OX375792.1	30	3.99	39.4
OX375762.1	Z	14.96	37
OX375793.1	MT	0.02	18

The estimated Quality Value (QV) of the final assembly is 65.8 with *k*-mer completeness of 100%, and the assembly has a BUSCO v5.3.2 completeness of 98.4% (single = 98.0%, duplicated = 0.4%), using the lepidoptera_odb10 reference set ( *n* = 5,286).

Metadata for specimens, spectral estimates, sequencing runs, contaminants and pre-curation assembly statistics can be found at https://links.tol.sanger.ac.uk/species/934925.

## Methods

### Sample acquisition and nucleic acid extraction

A male *Chesias legatella* (specimen number NHMUK014543814, ToLID ilCheLega1) was collected from Beinn Eighe National Nature Reserve, Scotland, UK (latitude 57.63, longitude –5.35) on 10 September 2021. The specimen was collected by David Lees (Natural History Museum) using a light trap. The specimen was identified by the collector and preserved at –80°C. 

The ilCheLega1 sample was weighed and dissected on dry ice with tissue set aside for Hi-C sequencing. Head and thorax tissue of ilCheLega1 was disrupted using a Nippi Powermasher fitted with a BioMasher pestle. DNA was extracted at the Wellcome Sanger Institute (WSI) Scientific Operations core using the Qiagen MagAttract HMW DNA kit, according to the manufacturer’s instructions.

### Sequencing

Pacific Biosciences HiFi circular consensus DNA sequencing libraries were constructed according to the manufacturers’ instructions. DNA sequencing was performed by the Scientific Operations core at the WSI on Pacific Biosciences SEQUEL II (HiFi) instrument. Hi-C data were also generated from tissue of ilCheLega1 using the Arima v2 kit and sequenced on the Illumina NovaSeq 6000 instrument.

### Genome assembly, curation and evaluation

Assembly was carried out with Hifiasm ( Cheng *et al.*, 2021) and haplotypic duplication was identified and removed with purge_dups ( Guan *et al.*, 2020). The assembly was scaffolded with Hi-C data ( Rao *et al.*, 2014) using YaHS ( Zhou *et al.*, 2023). The assembly was checked for contamination as described previously ( Howe *et al.*, 2021). Manual curation was performed using HiGlass ( Kerpedjiev *et al.*, 2018) and Pretext ( Harry, 2022). The mitochondrial genome was assembled using MitoHiFi ( Uliano-Silva *et al.*, 2022), which performed annotation using MitoFinder ( Allio *et al.*, 2020). To evaluate the assembly, MerquryFK was used to estimate consensus quality (QV) scores and *k*-mer completeness ( Rhie *et al.*, 2020). The genome was analysed and BUSCO scores ( Manni *et al.*, 2021; Simão *et al.*, 2015) were calculated within the BlobToolKit environment ( Challis *et al.*, 2020). Table 3 contains a list of software tool versions and sources.

**Table 3.** Software tools: versions and sources.

Software tool	Version	Source
BlobToolKit	4.0.7	https://github.com/blobtoolkit/blobtoolkit
BUSCO	5.3.2	https://gitlab.com/ezlab/busco
Hifiasm	0.16.1-r375	https://github.com/chhylp123/hifiasm
HiGlass	1.11.6	https://github.com/higlass/higlass
Merqury	MerquryFK	https://github.com/thegenemyers/MERQURY.FK
MitoHiFi	2	https://github.com/marcelauliano/MitoHiFi
PretextView	0.2	https://github.com/wtsi-hpag/PretextView
purge_dups	1.2.3	https://github.com/dfguan/purge_dups
YaHS	yahs-1.1.91eebc2	https://github.com/c-zhou/yahs

### Ethics and compliance issues

The materials that have contributed to this genome note have been supplied by a Darwin Tree of Life Partner. The submission of materials by a Darwin Tree of Life Partner is subject to the Darwin Tree of Life Project Sampling Code of Practice. By agreeing with and signing up to the Sampling Code of Practice, the Darwin Tree of Life Partner agrees they will meet the legal and ethical requirements and standards set out within this document in respect of all samples acquired for, and supplied to, the Darwin Tree of Life Project. All efforts are undertaken to minimise the suffering of animals used for sequencing. Each transfer of samples is further undertaken according to a Research Collaboration Agreement or Material Transfer Agreement entered into by the Darwin Tree of Life Partner, Genome Research Limited (operating as the Wellcome Sanger Institute), and in some circumstances other Darwin Tree of Life collaborators.

## Data Availability

European Nucleotide Archive: *Chesias legatella*. Accession number PRJEB55725; https://identifiers.org/ena.embl/PRJEB55725. ( Wellcome Sanger Institute, 2022) The genome sequence is released openly for reuse. The *Chesias legatella* genome sequencing initiative is part of the Darwin Tree of Life (DToL) project. All raw sequence data and the assembly have been deposited in INSDC databases. The genome will be annotated using available RNA-Seq data and presented through the Ensembl pipeline at the European Bioinformatics Institute. Raw data and assembly accession identifiers are reported in Table 1.

## Author information

Members of the Natural History Museum Genome Acquisition Lab are listed here: https://doi.org/10.5281/zenodo.4790042.

Members of the Darwin Tree of Life Barcoding collective are listed here: https://doi.org/10.5281/zenodo.4893703.

Members of the Wellcome Sanger Institute Tree of Life programme are listed here: https://doi.org/10.5281/zenodo.4783585.

Members of Wellcome Sanger Institute Scientific Operations: DNA Pipelines collective are listed here: https://doi.org/10.5281/zenodo.4790455.

Members of the Tree of Life Core Informatics collective are listed here: https://doi.org/10.5281/zenodo.5013541.

Members of the Darwin Tree of Life Consortium are listed here: https://doi.org/10.5281/zenodo.4783558.
