# Supplementary material for: The genome sequence of the Streak, Chesias legatella (Denis & Schiffermüller, 1775)
Source: Wellcome Open Res. 2024 Sep 3;8:205. Originally published 2023 May 10. [Version 2] doi: 10.12688/wellcomeopenres.19298.2 (PMC11377919; doi:10.12688/wellcomeopenres.19298.2)
Supplement: Peer review history

# Reviewer report: Reviewer response for version 2

**DOI:** 10.21956/wellcomeopenres.24720.r96944

**Published:** 2024-09-05

**Authors:** Kay Lucek

**Affiliations:** 1 Department of Environmental Sciences, University of Neuchâtel, Neuchâtel,, Switzerland

I appreciate clarification concerning the annotation.

Are sufficient details of methods and materials provided to allow replication by others?

Partly

Is the rationale for creating the dataset(s) clearly described?

Yes

Are the datasets clearly presented in a useable and accessible format?

Partly

Are the protocols appropriate and is the work technically sound?

Yes

Reviewer Expertise:

Speciation, Genomics, Evolutionary Biology, Lepidoptera

I confirm that I have read this submission and believe that I have an appropriate level of expertise to confirm that it is of an acceptable scientific standard.

# Reviewer report: Reviewer response for version 2

**DOI:** 10.21956/wellcomeopenres.24720.r96946

**Published:** 2024-09-05

**Authors:** Fahad Alqahtani

**Affiliations:** 1 King Abdulaziz City for Science and Technology, Riyadh, Saudi Arabia

I am happy with the manuscript in its current form.

Are sufficient details of methods and materials provided to allow replication by others?

Yes

Is the rationale for creating the dataset(s) clearly described?

Yes

Are the datasets clearly presented in a useable and accessible format?

Yes

Are the protocols appropriate and is the work technically sound?

Yes

Reviewer Expertise:

Bioinformatics

I confirm that I have read this submission and believe that I have an appropriate level of expertise to confirm that it is of an acceptable scientific standard.

# Reviewer report: Reviewer response for version 1

**DOI:** 10.21956/wellcomeopenres.21381.r71927

**Published:** 2024-03-01

**Authors:** Shota Nomura

**Affiliations:** 1 Division of Evolutionary Developmental Biology, National Institute for Basic Biology, Okazaki, Japan

This study performed the chromosome-level genome assembly of the Streak, *Chesias legatella*. Authors assembled the contigs using PacBio single-molecule HiFi long reads and scaffolded them using Hi-C data. As a result, the authors obtained 36 scaffolds with 11.0 Mb of N50 and 98.4% of BUSCO score. Among the assembly, 99.95% were assigned to 30 autosomes and the Z sex chromosomes, resulting in a chromosome-level genome assembly with very high completeness.

 In this manuscript, the methods of analyses were appropriate and well explained. I note below a few minor questions.

 1. Authors wrote “the whole genome will be useful for more detailed evolutionary studies”. How many species of the same genus or tribe have chromosome-level genome sequences been published? This information would be useful to readers who wants to perform evolutionary studies and would be better to be explained in the paragraph.

 2. As well as other papers submitted to Wellcome open research, genome annotation analyses should be performed using BRAKER2 or others pipeline. Annotation information is very useful to readers who use the assembled genome sequences for other studies. Also, the statement in Data Availability section (“The genome will be annotated using available RNA-Seq data and presented through the Ensembl pipeline at the European Bioinformatics Institute”) should be modified.

 3. How did authors distinguish Z sex chromosomes from autosomes? If authors obtained male and female reads, they can be distinguished based on differences in reads mapping between the sexes. Did the authors use such a method? In any case, it would be better to be explained the methods of distinguish them.

 4. “Hi-C data” should be changed to “Hi-C library” in Sequencing paragraph in Method section.

Are sufficient details of methods and materials provided to allow replication by others?

Partly

Is the rationale for creating the dataset(s) clearly described?

Yes

Are the datasets clearly presented in a useable and accessible format?

Partly

Are the protocols appropriate and is the work technically sound?

Yes

Reviewer Expertise:

Evolutionary ecology, genomics, RNA-seq, insect, Carabidae, speciation, sexual selection

I confirm that I have read this submission and believe that I have an appropriate level of expertise to confirm that it is of an acceptable scientific standard, however I have significant reservations, as outlined above.

# Reviewer report: Reviewer response for version 1

**DOI:** 10.21956/wellcomeopenres.21381.r75002

**Published:** 2024-02-26

**Authors:** Fahad Alqahtani

**Affiliations:** 1 King Abdulaziz City for Science and Technology, Riyadh, Saudi Arabia

This paper is about the genome sequence of the Streak, Chesias legatella (Denis & Schiffermüller, 1775), the authors report that they successful reconstructed the genome at the chromosomal-level for a male specimen of Chesias legatella. They utilized two sequencing technologies, Pacific Biosciences SEQUEL II and Hi-C Illumina, to assemble the genome, which is approximately 310.3 Mb in size. The completeness of the genome assembly was assessed with BUSCO analysis, which contains 98.4% of common genes were completely present.

 However, minor comments should be addressed:

 - The photo of the Streak, Chesias legatella in Figure 1 needs to be imporoved.

 - The NCBI-BioSample (SAMEA14448143) entry should be updated to reflect that the specimen is male, as stated in the paper.

 - It is recommended to mention the related species used for guiding the mitochondrial genome annotation with the Mitofinder tool in the methods section.

 - The term "Pretext" in the Genome Assembly section should be corrected to "PretextView".

Are sufficient details of methods and materials provided to allow replication by others?

Yes

Is the rationale for creating the dataset(s) clearly described?

Yes

Are the datasets clearly presented in a useable and accessible format?

Yes

Are the protocols appropriate and is the work technically sound?

Yes

Reviewer Expertise:

Bioinformatics

I confirm that I have read this submission and believe that I have an appropriate level of expertise to confirm that it is of an acceptable scientific standard, however I have significant reservations, as outlined above.

# Reviewer report: Reviewer response for version 1

**DOI:** 10.21956/wellcomeopenres.21381.r71925

**Published:** 2024-01-08

**Authors:** Kay Lucek

**Affiliations:** 1 Department of Environmental Sciences, University of Neuchâtel, Neuchâtel,, Switzerland

The genome sequence of *Chesias legatella* is providing great potential for future evolutionary studies. Importantly, the notion that this species could be used as an agent for biological control “probably without consequences” needs to be carefully assessed before such measures would be implemented.

 Overall the standard pipelines for genome assembly of the Darwin Tree of Life project have been thoroughly implemented.

 Although the data availability statement suggests that "available RNA-Seq data" will be used to annotate the genome, no information on how this dataset has been generated or information about its quality are provided.

Are sufficient details of methods and materials provided to allow replication by others?

Partly

Is the rationale for creating the dataset(s) clearly described?

Yes

Are the datasets clearly presented in a useable and accessible format?

Partly

Are the protocols appropriate and is the work technically sound?

Yes

Reviewer Expertise:

Speciation, Genomics, Evolutionary Biology, Lepidoptera

I confirm that I have read this submission and believe that I have an appropriate level of expertise to confirm that it is of an acceptable scientific standard, however I have significant reservations, as outlined above.

# Response

**Published:** 2024-05-11

**Authors:** Tree of Life Team Sanger

**Affiliations:** Wellcome Trust Sanger Institute, UK

Thank you for your comments on this data note.  We would like to clarify the statement in the Data availability section: "The genome will be annotated using available RNA-Seq data and presented through the Ensembl pipeline at the European Bioinformatics Institute." This is not intended to refer to RNA-Seq generated as part of the dataset presented here. We have not presented RNA data here, and the Ensembl Rapid Annotation pipeline uses RNA data from different sources. 

 We have added Ensembl annotation to the new version of this article, which was provided via their BRAKER2 pipeline. This annotation is generated externally to the Wellcome Sanger Institute.
